# Discriminating between viable and membrane-damaged cells of the plant pathogen *Xylella fastidiosa*

**DOI:** 10.1371/journal.pone.0221119

**Published:** 2019-08-23

**Authors:** Anne Sicard, Marcus V. Merfa, Michael Voeltz, Adam R. Zeilinger, Leonardo De La Fuente, Rodrigo P. P. Almeida

**Affiliations:** 1 Department of Environmental Science, Policy and Management, University of California, Berkeley, California, United States of America; 2 Department of Entomology and Plant Pathology, Auburn University, Auburn, Alabama, United States of America; Universidade do Minho, PORTUGAL

## Abstract

*Xylella fastidiosa* is a plant pathogenic bacterium with devastating consequences to several crops of economic importance across the world. While this pathogen has been studied for over a century in the United States, several aspects of its biology remain to be investigated. Determining the physiological state of bacteria is essential to understand the effects of its interactions with different biotic and abiotic factors on cell viability. Although *X*. *fastidiosa* is culturable, its slow growing nature makes this technique cumbersome to assess the physiological state of cells present in a given environment. PMA-qPCR, i.e. the use of quantitative PCR combined with the pre-treatment of cells with the dye propidium monoazide, has been successfully used in a number of studies on human pathogens to calculate the proportion of viable cells, but has less frequently been tested on plant pathogens. We found that the use of a version of PMA, PMAxx, facilitated distinguishing between viable and non-viable cells based on cell membrane integrity *in vitro* and *in planta*. Additional experiments comparing the number of culturable, viable, and total cells *in planta* would help further confirm our initial results. Enhancers, intended to improve the efficacy of PMAxx, were not effective and appeared to be slightly toxic to *X*. *fastidiosa*.

## Introduction

The pathogenic bacterium *Xylella fastidiosa* is associated with several economically important diseases of crop plants [1]. Although efforts have been allocated to study this pathogen at the time of outbreaks, the complex interactions of this bacterium with its various hosts as well as its complex biology have led to remaining gaps in knowledge. In addition, there are questions that require the development of new techniques. Real-time PCR, or quantitative PCR, has been used for almost two decades [2] to study aspects of *X*. *fastidiosa* biology as diverse as bacterial detection [3], multiplication within plant hosts [2–4], response to different minerals [5], the impact of a bacterial gene knockout on its multiplication within insect vectors [6], and correlation between *X*. *fastidiosa* population within insect vectors and transmission [7]. Although these studies are of importance to better understand *X*. *fastidiosa* interactions with different plant species and insect vectors, they do not inform the physiological state of the pathogen, primarily cell viability. Although the distinction between viable and dead cells is often overlooked, this understanding is epidemiologically critical.

As *X*. *fastidiosa* is culturable, several studies have used culturing to assess the presence of live bacterial cells in plant hosts (e.g. [8,9]) and to determine whether the bacterium was multiplying within hosts (e.g. [10,11]). Culturing is prone to contamination, especially in the case of this fastidious slow-growing plant pathogen, and samples cannot be stored for later processing. Depending on the strain, it can also take up to a few weeks to obtain results. Finally, cells can enter a persistence or “dormant” state, in which they have an intact cell membrane but are metabolically inactive [12–14].

The first studies to look at *X*. *fastidiosa* cell viability used a combination of DNA binding dyes Syto9/propidium iodide and fluorescent microscopy [15–17]. The nucleic acid intercalating dye, ethidium monoazide (EMA) coupled to qPCR has also been used in previous studies [5,18] to assess *X*. *fastidiosa* cell viability in vitro. After entering cells with damaged membranes, EMA binds to DNA in a covalent manner under light exposure preventing its amplification by qPCR. As a consequence, after EMA treatment, only DNA from cells with intact membranes is detected by qPCR. The population of non-treated samples, which corresponds to the whole population (dead and viable cells), and the population of EMA-treated samples–(viable cells)–thus enables determination of the population of cells with damaged membranes. However, EMA has been shown to penetrate viable cells, leading to an underestimation of their numbers [19,20]. Furthermore, these studies did not try to optimize the use of these reagents to discriminate between *X*. *fastidiosa* dead and viable cells. The use of the reagent propidium monoazide (PMA), which has a similar operating mode as EMA, has been tested successfully on several bacterial species (e.g. [19–21]) and fungi [22]. As opposed to EMA, PMA has not been reported to penetrate cells with intact membranes when used at low concentrations [19]. Therefore, it has become the method of choice to look at viable cell populations.

As *X*. *fastidiosa* represents an important threat to a number of crops in several regions of the world, developing a quick and easy way to look at cell viability would be useful in academic, regulatory, and quarantine contexts. We thus aimed to test the combination of a commercially available version of PMA, PMAxx, with qPCR on this bacterial species. Our objectives were to test the efficacy of PMAxx-qPCR to distinguish between viable and heat-killed *X*. *fastidiosa* cells, evaluate the utility of pre-treatments designed to improve the penetration of PMA in Gram-negative cells with damaged membranes, and to assess whether this method could be used *in planta* to estimate *X*. *fastidiosa* viable cell populations.

## Materials and methods

### Strains and media

*Xylella fastidiosa* subspecies *fastidiosa* STL and Temecula1 strains were propagated on periwinkle wilt (PW) medium [23] with or without Gelrite [24]. Cells were resuspended in either PD2 medium [25] or succinate-citrate-phosphate buffer (SCP, [26]), and the OD_600_ was standardized before conducting each *in vitro* assay.

### Plant bioassays

Four-week-old *Nicotiana tabacum* L. cv. Petit Havana SR1 plants, located at the Plant Science Research Center at Auburn University (AL, USA), were pinprick-inoculated with 15 μL of *X*. *fastidiosa* strain Temecula1 at a concentration of ~10^8^ CFU/mL. Leaf samples from 11 tobacco plants were taken 15 weeks post inoculation from greenhouse-inoculated plants and kept on ice until processed. The tobacco plants were heavily symptomatic at the sampling time. Leaves from healthy tobacco plants were also collected as control, and were proved to be negative for *X*. *fastidiosa* infection through qPCR.

In a similar manner, 2 months-old *Vitis vinifera* cv. Cabernet Sauvignon cuttings, located at the Oxford Tract Facility at the University of California, Berkeley (CA, USA) were inoculated with 20 μL of a *X*. *fastidiosa* strain STL suspension at a concentration of 10^8^ CFU/mL. Petioles from 12 and 17 grapes were sampled five and ten weeks post inoculation (wpi), respectively. At 10 wpi, the samples were harvested between 3.5 cm and 40 cm above the inoculation point. Only the beginning of marginal leaf scorching was observed at 5 wpi while a couple more advanced scorched leaves were observed at 10 wpi. Uninfected grapevine petioles were collected from *Vitis vinifera* cv. Cabernet Sauvignon from the Oxford Tract Facility and used as a negative control.

### Bacterial population estimation from inoculated plants

For tobacco samples, leaves were surface-cleaned using running water, and 150 mg of the petiole were taken from each leaf. Petioles were cut into pieces and ground in 2 mL microcentrifuge tubes with two 6.35 mm dia. chrome steel beads and 800 μL of PD2 broth (BioSpec Products, USA) in a Mini-Beadbeater (BioSpec Products, USA), and resuspended to a final volume of 4 mL using PD2 broth, prior to cell treatments and DNA extraction.

Both *X*. *fastidiosa* infected and uninfected grapevine samples were homogenized as previously described [24]. Briefly, grapevine petioles were weighted to 0.1 g before being sterilized in 30% bleach for 2 minutes, 70% ethanol for 2 minutes and three baths of distilled water for 1 minute each. Sterilized petioles were then cut with a razor blade and homogenized using a Polytron (Brinkman Instruments, Inc., Westbury, NY) in SCP buffer before being further processed. After homogenization, *X*. *fastidiosa* infected samples were serially diluted and plated on PWG following the same process as previously described to assess the number of Colony Forming Units (CFU, [24]). PWG plates were incubated for 10 days at 28°C before CFU were counted. In parallel, 1 mL of each infected plant homogenate was divided into two fractions of 500 μL each, which were analyzed using either qPCR or PMAxx-qPCR (see section “Cell treatments” for details) to determine the total and the viable cell populations respectively. The uninfected grapevine petioles were spiked with different percentages of viable (V) and heat-killed cells (H): (1) 0% V—100% H, (2) 25% V—75% H, (3) 50% V—50% H, (4) 75% V—25% H and (5) 100% V—0% H in order to test the accuracy with which PMAxx-qPCR detects live cells in a sample containing known amounts of live cells and plant cell debris.

### Cell treatments

For the *in vitro* experiments, *X*. *fastidiosa* cells were harvested from PW or PWG plates three days after having been replated to maximize the number of viable cells [5] and resuspended in SCP or PD2 broth. Each suspension was divided into several aliquots of 500 μL each in DNA LoBind microcentrifuge 1.5 mL tubes (Eppendorf) before being further processed. This was done to ensure that the differences between treatments could not be linked to variations of starting cell concentrations. Samples were always kept on ice when not handled. An aliquot of cells was killed by heating at 95°C for 20 minutes in a water bath and were vortexed 2–3 times during the heat treatment.

PMAxx (Biotium, Hayward, USA) was used at a final concentration of 50 μM unless otherwise stated, while the PMA Enhancer for Gram-negative Bacteria (Biotium, Hayward, USA; hereafter termed ‘enhancer’) was used at a final concentration of 1X, and sodium deoxycholate (DOC, Sigma-Aldrich) at 0.1%. After treatment, all samples were incubated for 10 min in the dark on a shaker before being exposed for 15 min on ice under agitation to either an LED (1500 lumen) or a halogen light (700 W) at a distance of 20 cm from the light source. Samples were then pelleted by centrifugation at 8,000 x g for 30 minutes and conserved at -20°C until DNA extraction. As a control, samples not treated with PMAxx were also incubated in the dark and exposed to light. An aliquot of cells from some treatments was also used to determine the culturable population by serial dilution plating on medium, as described above.

### DNA extraction and qPCR

DNA from *in vitro* experiments was extracted using either the DNeasy Blood and Tissue kit (Qiagen) or the ZR Fungal/Bacteria DNA Miniprep kit (ZYMO Research), which was used for the *in vitro* assays determining the role of DOC on cell viability. Both kits were used following the manufacturers’ instructions. For grapevine samples, the extraction was carried out using the DNeasy Plant Mini kit (Qiagen) while a modified CTAB protocol was used for tobacco samples [27]. qPCR was conducted as previously described [28] using primers designed for this study, PD0059 F+R (Table 1 and S1 Table)–designed near the origin of replication–at a concentration of 0.3 μM. For comparison, a subset of the samples was also analyzed using (i) HL5/HL6 primers at 0.2 μM, in combination or not with the HLP TaqMan probe labeled with FAM at a concentration of 0.15 μM (HL5/HL6/HLP, [28]), and (ii) PD1080 F+R–primers designed for this study in the same region as HL5/HL6 –used at a final concentration of 0.3 μM (see S1 Table for information on primers). Each sample was run in duplicate by qPCR. For each qPCR plate, fluorescence data were normalized using a reference dye (ROX), and analyzed with the LinRegPCR program [29]. Cell populations were determined using a standard curve of strains STL or Temecula1 as appropriate.

**Table 1:** Impact of primer efficiency on the Ct values and on the detected populations of total (live and “naturally dead”) or heat-killed bacteria. The cells have been either non-treated (NT) or PMAxx–treated (PMA).

Primer pair	Sample	Ct value	ΔCt	CFU/mL	Primer efficiency	Amplicon size	Genomic position (bp) ^a^
HL5/HL6	Total_NT	14.8±0.40		2.4*10^8^±3.2*10^7^	80.8%	221 bp	1,280,158–1,280,318
	Total_PMA	16.3±0.46		1.4*10^8^±2.3*10^7^			
	Heat killed_NT	19.8±0.13	16.5±1.3	4.5*10^7^±2.0*10^6^			
	Heat killed_PMA	36.3±1.35		2.0*10^5^±8.3*10^4^			
PD0059	Total _NT	11.9±0.28		3.0*10^8^ ±4.0*10^7^	94.2%	178 bp	78,318–78,493
	Total_PMA	12.7±0.22		1.9*10^8^±2.2*10^7^			
	Heat killed_NT	15.6±0.17	12.5±1	4.2*10^7^±4.0*10^6^			
	Heat killed_PMA	28.1+/-1.1		1.1*10^5^±5.8*10^4^			
PD1080	Total _NT	12.4±0.16		3.0*10^8^±2.3*10^7^	94.7%	122 bp	1,280,257–1,280,378
	Total_PMA	13.1±0.19		2.1*10^8^±1.9*10^7^			
	Heat killed_NT	16.4±0.09	11.8±1.2	4.6*10^7^±1.9*10^6^			
	Heat killed_PMA	28.2±1.28		2.0*10^5^±1.2*10^5^			

^a^ Genomic positions are based on the genome of the *X*. *fastidiosa* Temecula1 strain.

### Statistical analyses

We conducted linear regression or ANOVA tests as appropriate for each experiment. To meet assumptions of the linear models, estimated CFU/mL were transformed using log10, square root, or 4^th^ root based on Box-Cox tests. Likewise, C_t_ values were inverse square root transformed. Independently repeated trials for the same experiment were treated as block effects, similar to a randomized complete block design, except in some cases when the treatment-by-block interaction was not estimated because not all treatments were included in all blocks. Block terms were specified as fixed effects; preliminary analyses indicated that specifying them as random effects did not change the overall results. Statistical analyses were performed using R (Version 1.0.143). Further details on statistics are described within the text.

## Results

### Effect of PMAxx and pre-treatments on *X*. *fastidiosa* culturability and viability

**Fig 1:**
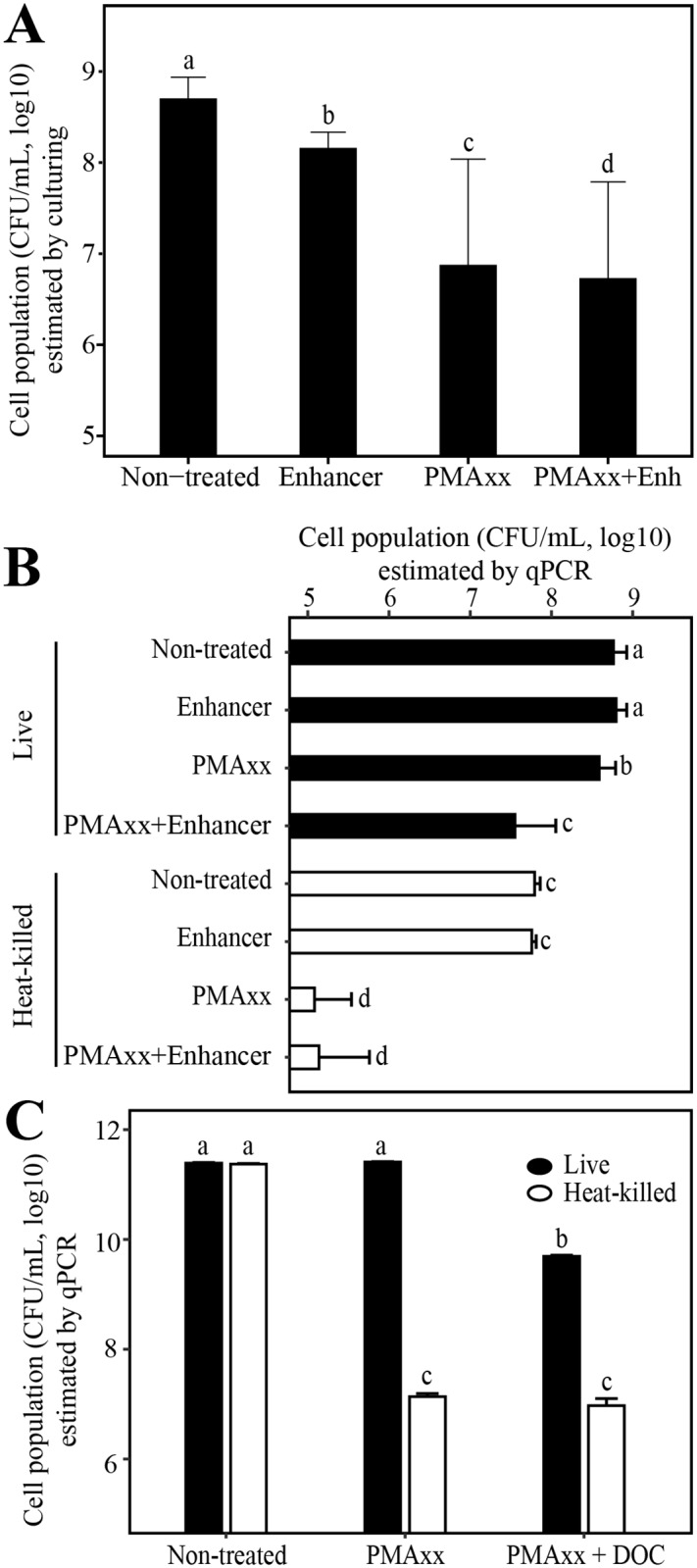
Effect of PMAxx, PMA Enhancer for Gram-negative Bacteria (Enhancer, Enh) and sodium deoxycholate (DOC) on *Xylella fastidiosa* cell viability and culturability. (A) PMAxx, enhancer, and a combination of both treatments significantly decreased the number of culturable cells. (B) When combined with the enhancer, PMAxx significantly decreased the number of viable cells detected by qPCR, but did not significantly decrease the number of dead still detected after PMAxx treatment. (C) Similarly, when using both DOC and PMAxx, the number of viable cells detected by qPCR was significantly impacted while the population of heat-killed cells remained unchanged compared to PMAxx alone. PMAxx does not impact the number of viable cells detected by qPCR. For (A) and (B), error bars indicate standard errors from two independent experiments consisting of four independent replicates each while in (C), error bars indicate the standard errors from three independent replicates.

To assess the effect of PMA reagents on cell culturability and determine whether they were toxic, cells were plated after treatment with PMAxx, enhancer, both PMAxx and enhancer, or in the absence of treatment. We used Tukey’s HSD to test for differences in the mean square-root transformed CFU/mL among treatments based on the family-wise 95% confidence intervals (CI), reported as [2.5% CI, 97.5% CI]; two treatment means are significantly different if the 95% CI do not overlap zero. Both the enhancer and PMAxx had a significant impact on cell culturability relative to non-treated cells (Tukey’s HSD; Non-treated–Enhancer = 8142 [4017, 12266], *P* = 1.5*10^−5^; Non-treated–PMAxx = 14855 [10730, 18980], *P* < 10^−7^). An even larger effect was noticed when these treatments were used in combination (Fig 1A).

To assess whether this impact on cell culturability was reflected in a DNA-based assay, we analyzed the same samples by qPCR. As expected, no difference was observed when comparing non-treated samples with samples treated with the enhancer alone as this treatment per se does not discriminate between viable and dead cells, hence leading to the detection of the total number of cells by qPCR (Tukey’s HSD; qPCR_Enhancer–qPCR_Non-treated = 426 [-1407, 2259, *P* = 0.99; Fig 1B). Although the total number of cells (non-treated) determined by qPCR was significantly different from the number of detected viable cells (Tukey’s HSD; qPCR-PMAxx—qPCR-non-treated = - 3268 [-5101, - 1435], *P* = 3.4*10^−6^), no difference could be detected between the number of viable cells (qPCR_PMAxx) and the number of culturable cells (Culturability_non-treated) based on CFU on solid media (ANOVA, *F*_*1*,*46*_ = 0.53, *P* = 0.47, mean_Culturability ± SE_Culturability = 5,36E+08 ± 2,53E+08 and mean_Viability ± SE_Viability = 4,75E+08 ± 2,16E+08). In other words, PMAxx treatment did not have a significant impact on the number of viable cells detected by qPCR, with all culturable cells being detected by PMAxx-qPCR. However, a combination of PMAxx and enhancer led to a significant decrease in the number of viable cells detected by qPCR (Tukey’s HSD; qPCR-non-treated–qPCR_PMAxx + Enhancer = 15610 [13777, 17443]; *P* < 10^−7^).

**Fig 2:**
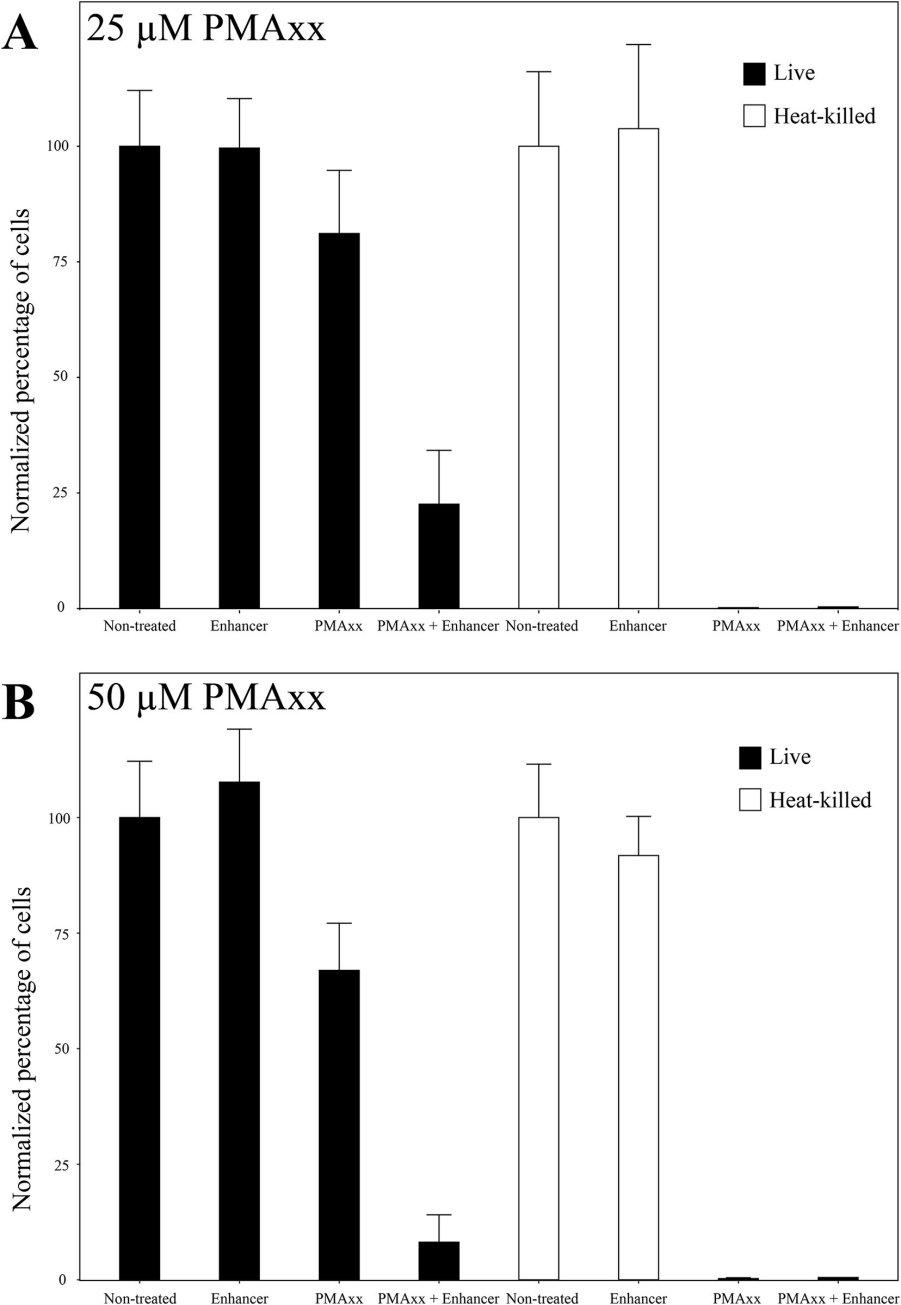
Percentage of viable and heat-killed cells detected by qPCR after treatment with PMAxx at a concentration of either (A) 25μM or (B) 50μM with and without the Enhancer. The percentage of cells was normalized by the mean concentration of total number of cells (live non-treated or heat-killed non-treated).

In parallel, samples were heat-killed and treated with the same reagents. No cell could be detected on plates after heat treatment except for two out of the fifteen replicates in which ~ 4000 CFU/mL in total were observed on PWG after heat treatment. As expected, no difference between the non-treated heat-killed cells and the ones treated with enhancer alone could be detected (Tukey’s HSD, qPCR_Non-treated–qPCR_Enhancer = 73 [-1907, 1759], *P* = 1). The treatment with PMAxx and the combination of the two reagents significantly decreased the number of detected cells (Tukey’s HSD, qPCR_PMAxx–qPCR_Non-treated = -3268 [-5101, -1435], *P* < 10^−7^ and qPCR_PMAxx + Enhancer–qPCR_Non-treated = -8808 [-10641, -6974], *P* = 3.4*10^−6^; Fig 1B). However, neither of these two treatments led to the elimination of the signal; on average 0.23% +/- 0.19% of cells were still detected after these treatments (Fig 2). No difference was observed between heat-killed cells treated with PMAxx alone or in combination with enhancer (Tukey’s HSD, qPCR_PMAxx—qPCR_PMAxx + Enhancer = -116 [-1949, 1717]; *P* = 1). Since the addition of the enhancer has a negative impact on cell culturability and cell viability, while not decreasing the signal from heat-killed cells in comparison to the use of PMAxx alone, this pre-treatment seemed to be counterproductive in the discrimination between viable and dead cells. These experiments were performed with two different concentrations of PMAxx, 25 μM and 50 μM; culturability was significantly lower at the 50 μM concentration than at 25 μM (ANOVA, *F*_*1*,*23*_ = 27.05, *P* < 0.001), but cell viability showed no difference as estimated by qPCR (ANOVA, *F*_*1*,*122*_ = 0.48, *P* = 0.51).

In a similar manner, another pre-treatment for Gram-negative bacteria (DOC) was used to determine whether it could help better discriminate between *X*. *fastidiosa* viable and heat-killed cells. Pre-treatment with DOC before the addition of PMAxx had a significant and negative impact on the number of viable cells (Tukey’s HSD, qPCR_PMAxx + DOC—qPCR_Non-treated = -425094 [-436509, -413679], *P* < 10^−7^) without reducing the signal from heat-killed cells in comparison with PMAxx alone (Tukey’s HSD, qPCR_PMAxx + DOC—qPCR_PMAxx = -620 [-12036, 10795], *P* = 1; Fig 1C).

### Effect of PMAxx concentration, primer pairs, and light source

**Fig 3:**
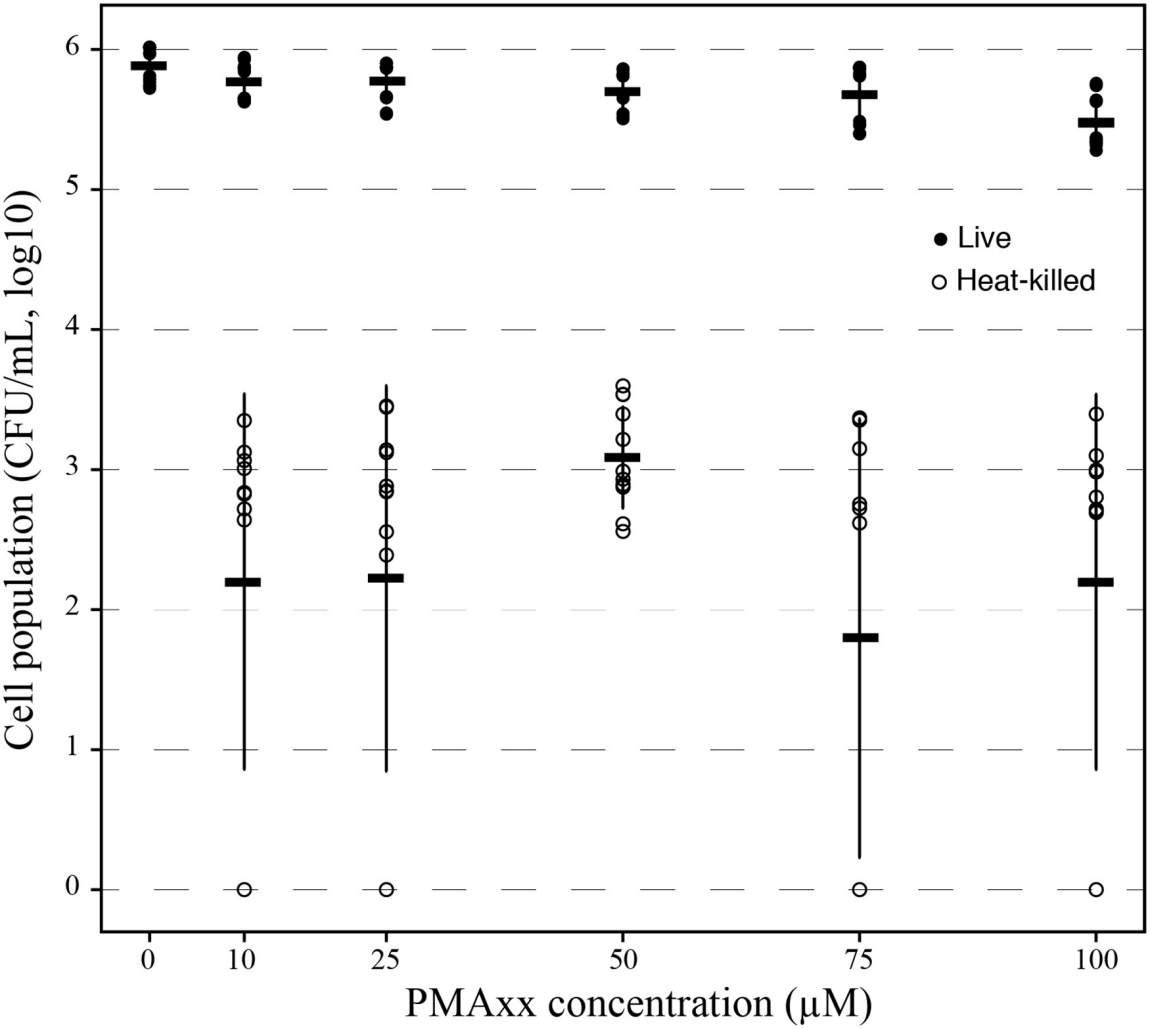
Optimization of PMAxx concentration for *X*. *fastidiosa*. Solid circles correspond to viable PMAxx treated cells and empty circles correspond to heat-killed cells still detected after PMAxx treatment by qPCR. The mean and standard deviation are from two experiments performed with three independent replicates each.

Different concentrations of PMAxx were tested to determine what concentration led to the best discrimination between *X*. *fastidiosa* viable cells and those with damaged membranes. The same suspension was divided into several aliquots that were heat-killed or not before being treated with a range of PMAxx concentrations. No significant differences between live and heat-killed cells were observed between 10 μM and 75 μM of PMAxx concentrations (Tukey’s HSD, *P* = 0.18), whereas a concentration of 100 μM significantly impacted this difference notably by decreasing the population of viable cells (Tukey’s HSD, *P* < 0.01, Fig 3). Again, on average 0.19% +/-0.15% of heat-killed cells remained detectable after PMAxx treatment whatever the concentration used. An increase of temperature (up to 105°C) or of time length (up to 50 min) to damage cell membranes did not help removing signal from PMA heat-killed samples (S2 Table).

To test whether qPCR primers could impact and help better distinguish between viable cells and cells with damaged membranes, the same samples were tested with three different primer pairs, including HL5/HL6 designed by Francis and coworkers [28]. Primer pairs had a significant impact on the Ct values (ANOVA, *F*_*2*,*84*_ = 827.64, *P* < 0.0001). Ct values for HL5/HL6 were always higher whatever samples tested (i.e. whether the cells were viable, dead, treated with PMAxx or non-treated) than when using either PD0059 F+R or PD1080 F+R (Table 1), the latest amplifying an amplicon in the same region as HL5/HL6. These differences of Ct values led to the absence of signal from heat-killed cells treated with PMAxx when run with either HL5/HL6 or HL5/HL6/HLP, while these samples were still amplified when tested with PD0059 F+R (see Ct values in Table 1). To determine whether the difference of Δ Ct values–between heat-killed cells non-treated and PMAxx-treated–observed between the different primer pairs were due to the difference of amplicon size or primer pair efficiency, both factors were tested by ANOVA. No effect of the amplicon size on Δ Ct was observed (estimate ± SE = 0.00891 ± 0.012, *t* statistic = 0.743, *P* = 0.47) while the efficiency of the primer pair had a significant impact (estimate ± SE = -13.6 ± 3.78, *t* statistic = -3.60, *P* = 0.0017). No significant difference was observed between PD1080 F+R and PD0059 F+R when considering the same treatment. While Ct values were different between primer pairs, and more specifically between HL5/HL6 and the two other primer pairs, no difference of cell population estimates was observed between primer pairs for a given treatment, meaning that the cell populations were correctly estimated by all primer pairs but led to a later amplification when using HL5/HL6 (Table 1). Finally, the impact of the light source for PMA treatment was tested using two different sources of light, a LED or a halogen lamp. No significant difference was observed (ANOVA, *F*_*1*,*104*_ = 1.59, *P =* 0.21, S3 Table).

### Test of PMAxx *in planta*

**Fig 4:**
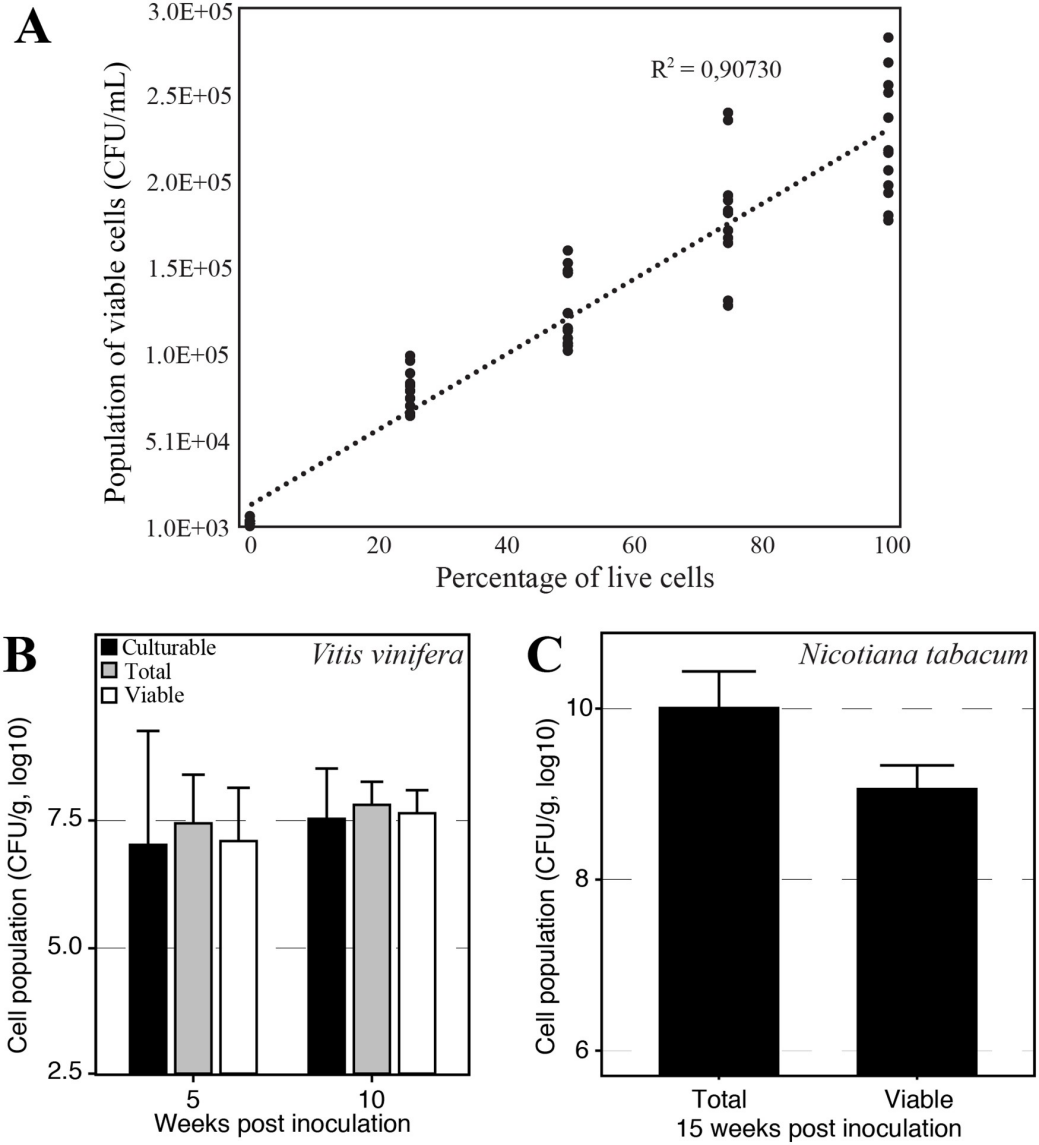
Test of PMAxx treatment on plant samples. (A) Uninfected *Vitis vinifera* petioles were spiked with different amounts of *X*. *fastidiosa* live cells before being further processed and treated with PMAxx. Data from two experiments consisting of three independent replicates each (and run by qPCR in duplicate) are shown here. (B) Test of PMAxx treatment on *X*. *fastidiosa* infected *Vitis vinifera* petioles sampled at 5 and 10 weeks post inoculation. (C) Test of PMAxx treatment on *X*. *fastidiosa* infected *Nicotiana tabacum* petioles sampled at 15 weeks post inoculation.

To test whether PMAxx could be used *in planta* to distinguish between *X*. *fastidiosa* viable and dead cells, we spiked uninfected grapevine petioles with different proportions of live and heat-killed bacterial cells before treating the samples with PMAxx. We obtained a strong correlation between the percentage of live cells present in the sample and the population of cells detected using PMAxx-qPCR (R^2^ = 0.93 and 0.94 for experiments one and two respectively, R^2^ = 0.9 when pooled, Fig 4A).

We sampled petioles from *X*. *fastidiosa*-infected grapevines five and ten weeks post inoculation. All the samples were cultured and analyzed by qPCR in parallel (non-treated samples, total number of cells) and PMAxx-qPCR (treated with PMAxx, viable cells). No significant difference was observed between the total number of cells, the number of viable cells and the number of culturable cells for both time points (ANOVA, *F*_*2*,*114*_ = 1.157, *P =* 0.32) while there was a significant effect of the sampling time on the cell concentration (ANOVA, *F*_*1*,*114*_ = 4.663, *P =* 0.033, Fig 4B). A negative correlation was observed between the number of total or viable cells and the distance to the inoculation point at ten weeks post inoculation (ANCOVA, *t* = -2.74, *P* = 0.01).

A similar experiment was performed using tobacco petioles sampled 15 weeks post inoculation. The total number of cells was significantly different from the number of viable cells (ANOVA, *F*_*1*,*64*_ = 113.7, *P =* 7.92e-16, Fig 4C). We found that on average 13.9% +/- 5.8% of the cells were still viable at the sampling time. The samples were not plated to assess culturability in parallel.

## Discussion

This is the first study looking at the use of PMAxx and PMA pre-treatments for the phytopathogen *X*. *fastidiosa*. Throughout this study, all *in vitro* experiments were performed three days after replating to maximize the proportion of culturable and viable cells [5]. We used both qPCR and culturing to better assess and understand the effects of PMAxx and pre-treatments designed for Gram-negative bacteria on *X*. *fastidiosa* cell viability and culturability. We demonstrate that neither of the pre-treatments, i.e. enhancer and DOC, reduced the qPCR signal coming from heat-killed cells treated with PMAxx, while both had a toxic effect on live cells. Sodium deoxycholate is a mild detergent known to affect cell membrane permeability [30,31] potentially leading to the penetration of PMAxx inside those cells. The mechanism by which the enhancer is partially toxic to cells remains unknown, as its composition has not been disclosed; but in regards to our results, it might impact the integrity or the permeability of *X*. *fastidiosa* cell membranes.

Although PMA was described as not being toxic to cells as opposed to EMA [19,20,32], we found that PMAxx significantly decreased the number of culturable *X*. *fastidiosa* cells without having a significant effect on the number of viable cells detected by qPCR at the concentration used in this study. This suggests that PMAxx has a toxic effect on *X*. *fastidiosa* cells leading to a decrease in their culturability. However, since the number of viable cells detected by qPCR was not altered, PMAxx does not appear to penetrate and bind DNA from the portion of cells impacted by this toxicity when used at 50 μM or less. Consequently, PMAxx treatment did not appear to underestimate the number of viable cells as opposed to EMA at the concentration used. Nonetheless, there was a significant decrease of the viable cell population when PMAxx was used at 100 μM. Underestimation of viable cell populations have already been reported for other bacterial species when using either high concentrations of PMA and/or lower amounts of targets (e.g. [33,34]). In addition, pre-treatment with DOC or the enhancer might lead to damaged cell membranes or changes in membrane permeability, which when followed by PMAxx treatment, leads to a reduction in the number of viable cells detected by qPCR.

We used heat treatment to kill cells and damage cell membranes to test the effectiveness of the PMAxx treatment. Heat is known to disrupt the integrity of cell wall and cell membrane by denaturing the constitutive proteins and rendering the phospholipids more fluid [34]. As a consequence, we expected all of the heat-killed cells to have damaged membranes and be susceptible to PMAxx. We observed throughout the study that a small percentage (~ 0.2% on average) of heat-killed cells had amplified DNA after PMAxx treatment, contrary to our expectations. This discrepancy could be due to the fact that a small percentage of cells still had an intact membrane even after heat treatment as it has been previously shown [34], and/or that PMAxx treatment does not lead to a complete reduction of qPCR signal from cells with damaged membranes that have been shown for a number of bacterial species when using PMA (e.g. [20,34–37]). As some cells were still culturable after heat treatment in two of the replicates, it seems likely that heat treatment does not kill all cells, thus preventing us from drawing a firm conclusion on the ability of PMAxx to completely discriminate between viable and *X*. *fastidiosa* cells with damaged membranes. Although each replicate corresponded to a single suspension divided into several aliquots before treatment, we observed in most of our experiments that the concentration of heat-killed cells non-treated was around 10-fold lower than the concentration of “live” non-treated cells. This difference–which had also been reported for *Salmonella* serovar *Enteridis* [34]—could be due to a partial degradation of DNA due to a prolonged exposition to heat or to a loss of DNA freed by heat treatment. Nonetheless, this was taken into account in the analyses.

When uninfected grapevine petioles were spiked with increasing numbers of *X*. *fastidiosa* live cells, a robust correlation was obtained between the number of live cells used and the number of viable cells detected by PMAxx-qPCR, leading towards the possibility to use this tool for *in planta* experiments. Petioles from *X*. *fastidiosa-*infected grapevines were then sampled at two different times post inoculation to test this hypothesis. No difference was detected between the total number of cells and the viable cells preventing us to draw a conclusion. These results are not surprising since the samples showed at most mild symptoms at the sampling times, and the fraction of blocked vessels is very small (0.053%) in asymptomatic grape leaves [38], suggesting that most cells were probably still alive, as observed by Chatterjee and coworkers in less heavily colonized vessels [15]. On the contrary, a significant difference was observed between the total number of cells and the number of viable cells in tobacco petioles at 15 weeks post inoculation, suggesting that a high proportion of cells were dead at this time of the infection. According to our results, on average, 86.1% +/- 5.8% of the cells had damaged cell membranes. As the plants were heavily symptomatic at this late stage of infection, this result is not unexpected, illustrating the potential usefulness of this approach.

No *in vitro* experiments were run to test whether PMAxx was effective at discriminating between *X*. *fastidiosa* viable cells and cells with damaged membranes present within biofilms. However, our results on tobacco petioles sampled at a late stage of infection–where most cells should be embedded within mature biofilms [15]–suggest that PMAxx-qPCR can be successfully applied to *X*. *fastidiosa* biofilms.

The side-by-side use of three different primer pairs shed some light on the importance of this variable on qPCR results. HL5/HL6 with or/without the HLP probe led to a complete absence of signal from PMAxx treated heat-killed cells even when working with a relatively high number of cells. However, when running the same samples with two other primer pairs, we noticed that the higher difference of Δ Ct value between heat-killed non-treated samples and PMAxx-treated ones were due neither to the amplicon size nor to the genomic region targeted, but to the inefficiency of HL5/HL6. This primer pair has low efficiency and should not be used for qPCR tests as an efficiency over 90% is generally recommended. Our results show that when starting with a lower amount of target–in this case corresponding to heat-killed PMAxx treated samples–there was very late or no amplification of target DNA in samples. This can be particularly problematic when working with plant samples or insect samples such as spittlebugs where the number of *X*. *fastidiosa* cells is small [7,39]. For example, using HL5/HL6 in this case would lead to a high number of false negative insects.

Based on these results, we recommend the use of PMAxx-qPCR without enhancers to determine *X*. *fastidosa* cell viability *in vitro*. Our results also point towards the ability of PMAxx to discriminate between viable and dead cells in plants; the use of PMAxx *in planta* remains nevertheless to be further tested. Although a concentration of 50 μM of PMAxx was mainly used throughout this study, our results show that a concentration as low as 10 μM can be used for *X*. *fastidiosa* to discriminate between viable cells and cells with damaged membranes. Furthermore, low concentrations of PMAxx might avoid toxic effect of this reagent when lower amounts of targets are present within the sample [34]. The confirmation of the ability of PMAxx-qPCR to accurately detect the viable cell population *in planta* would enable to further test the effect of several factors such as plant resistance and temperature on *X*. *fastidiosa* cell viability [40–42].

## Supporting information

S1 Table Sequences and concentrations of the primer pairs used in this study. (TIF)

S2 Table Effect of an increase of incubation time or temperature on *X*. *fastidiosa* cell populations. (TIF)

S3 Table Effect of light source on *X*. *fastidiosa* cell populations. The LED and halogen experiments were run separately for technical reasons; therefore, the starting cell populations were not exactly the same, and that difference is reflected in the results.
(TIF)
