# Supplementary figures and images for: Discriminating between viable and membrane-damaged cells of the plant pathogen Xylella fastidiosa
Source: PLoS One. 2019 Aug 23;14(8):e0221119. doi: 10.1371/journal.pone.0221119 (PMC6707623; doi:10.1371/journal.pone.0221119)

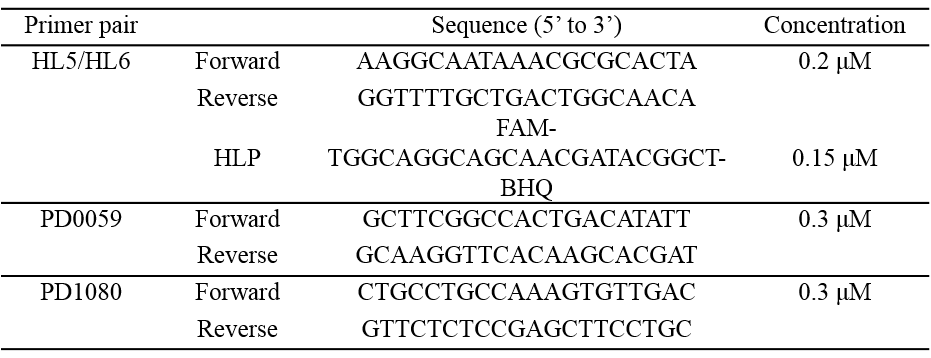

Supplement: S1 Table — (TIF) [file pone.0221119.s001.tif]

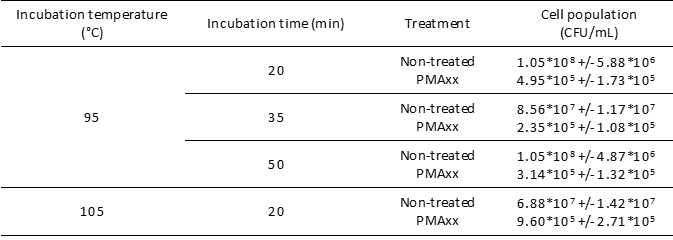

Supplement: S2 Table — (TIF) [file pone.0221119.s002.tif]

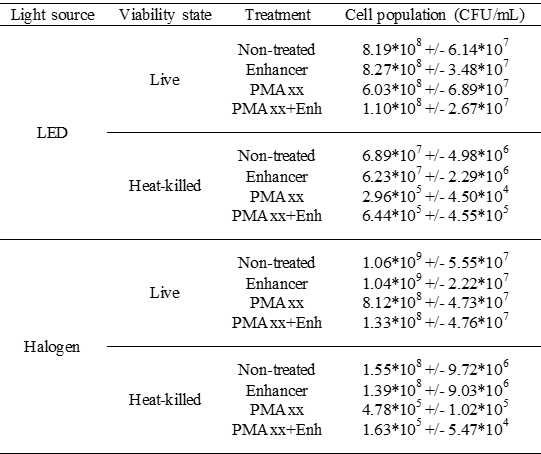

Supplement: S3 Table — The LED and halogen experiments were run separately for technical reasons; therefore, the starting cell populations were not exactly the same, and that difference is reflected in the results. (TIF) [file pone.0221119.s003.tif]
